# Silkworm Hemolymph Down-Regulates the Expression of Endoplasmic Reticulum Chaperones under Radiation-Irradiation

**DOI:** 10.3390/ijms12074456

**Published:** 2011-07-08

**Authors:** Kyeong Ryong Lee, Seung-Whan Kim, Young Kook Kim, Kisang Kwon, Jong-Soon Choi, Kweon Yu, O-Yu Kwon

**Affiliations:** 1 Department of Emergency Medicine, Konkuk University Medical Center, Seoul 143-729, Korea; E-Mail: lkrer@kuh.ac.kr; 2 Department of Emergency Medicine, Chungnam National University Hospital, Taejon 301-721, Korea; E-Mail: emfire@cnuh.co.kr; 3 Department of Social Spots, Joongbu University, Chungnam 312-702, Korea; E-Mail: yogkri@joongbu.ac.kr; 4 Department of Anatomy, College of Medicine, Chungnam National University, Taejon 301-747, Korea; E-Mail: ppkisang@empas.com; 5 Division of Life Science, Korea Basic Science Institute, Taejon 305-333, Korea; E-Mail: jschoi@kbsi.re.kr; 6 Graduate School of Analytical Science and Technology, Chungnam National University, Taejon 660-758, Korea; 7 Aging Research Center, Korea Research Institute of Bioscience and Biotechnology, Taejon 305-806, Korea; E-Mail: kweonyu@kribb.re.kr

**Keywords:** radiation, endoplasmic reticulum (ER), chaperone, silkworm hemolymph, ischemia-responsive protein 94 (irp94)

## Abstract

We demonstrated that up-regulation of gene expression of endoplasmic reticulum (ER) chaperones (BiP, calnexin, calreticulin, ERp29) and ER membrane kinases (IRE1, PERK, ATF6) was induced by radiation in neuronal PC12 cells. However, addition of silkworm, *Bombyx mori*, hemolymph to irradiated cells resulted in an obvious decrease in expression of these genes, compared with a single radiation treatment. In contrast, one of the ER chaperones, “ischemia-responsive protein 94 kDa” (irp94), was up-regulated by radiation. However, addition of silkworm hemolymph resulted in no change in the expression of irp94, with an expression pattern that differed from that of ER chaperones. Based on these results, we propose that silkworm hemolymph contains factors that regulate a decrease in the expression of ER chaperones under radiation-irradiation conditions, with the exception of irp94, which is not down-regulated. We suggest that this difference in the molecular character of irp94 may provide a clue to the biological functions associated with ER stress pathways, particularly the effects of radiation.

## 1. Introduction

The endoplasmic reticulum (ER), a eukaryotic organelle, is the location of post-translational modification. The ER is a major signal transducing organelle that senses and responds to changes in cellular homeostasis. ER stress can be induced by an unfolded protein response (UPR) or by viral infection (ER overload response, EOR), which mediates multiple molecular biological processes via ER stress sensors (inositol-requiring enzyme 1, IRE1, protein kinase-like ER kinase, PERK, and activating transcription factor 6, ATF6), which participate directly or indirectly in the UPR of mammalian cells [1–3]. The ER stress response in mammalian cells is triggered by dissociation of BiP from stress transducers, such as PERK, ATF6, and IRE1. BiP binds to ER lumenal un/misfolded proteins and activates the ER stress response [4]. The first response of ER stress involves up-regulation of genes encoding ER chaperones, which increase protein-folding/assembly activity and prevent protein aggregation in the ER. However, newly synthesized soluble and integral membrane secreted proteins are sometimes retained in the ER due to non-native conformations and are degraded by a process called ER-associated degradation (ERAD) [5]. Retention of a large number of non-native proteins sometimes induces ER storage disease (ERSD) [6]. Three functionally different groups of ER molecular chaperones reside in the ER: chaperones of the heat shock protein family, including BiP and its co-chaperone partners, chaperone lectins, such as calnexin and calreticulin, and the foldase family of PDI and ERp29. Radiation is known to induce a series of biochemical events in the cell, but whether radiation directly induces ER stress remains unclear. Recent reports have demonstrated that radiation induces expression of ERp29, a type of ER-resident chaperone, in several types of cultured cells [7]. UPR is rapidly sensitive to environmental or physical changes. However, incessant ER stressors induce the proapoptotic potential of the UPR, and eventually initiate apoptosis through the ER signal pathway [8].

Hemolymph is the circulating fluid of insects, similar to mammalian blood, which moves through the open circulatory system, directly bathing the organs and tissues. Compared with mammalian blood, insect hemolymph differs in the absence of erythrocytes and has a high concentration of several types of free amino acids. It serves important roles in the immune system and in transport of hormones, nutrients, and metabolites. Since the early 1970s, proteins of silkworm (*Bombyx mori*) hemolymph have been studied as part of the effort to produce more silk; thus, silkworm hemolymph is the most well-understood insect hemolymph. Cultured insect cells have demonstrated greater resistance to radiation-irradiation than those of mammals, and the lepidopteran cell, by far, shows the greatest resistance [9,10]; however, the detailed processes underlying this resistance have remained largely unexplored. Recent results have demonstrated that silkworm hemolymph inhibits apoptosis, and associated proteins were isolated [11–14].

The PC12 cell line used in this study is widely used as an *in vitro* model for the study of neuronal differentiation [15]. Several studies of PC12 differentiation have demonstrated an association of ionizing radiation with increased expression of cytokines [16], including IL-6 [17], through NFxB activation [18].

We are interested in the effect of silkworm hemolymph on gene expression of ER chaperones under radiation-irradiation in PC12 cells. In particular, we are interested in the expression of the “ischemia-responsive protein 94 kDa” (irp94) gene, which was first isolated from rat hippocampus. irp94, a novel member of the HSP110 family, was suggested to play an important role in transient forebrain ischemia [19]. We also demonstrated up-regulation of irp94 by ER stressors in PC12 cells [20,21]. Eventually, we would like to determine whether radiation-irradiation induces expression of ER chaperones, and how silkworm hemolymph affects gene expression under such conditions. Additionally, we would like to determine whether the molecular behavior of irp94 to both radiation-irradiation and silkworm hemolymph is the same as those of ER chaperones in the PC12 cell. This may provide clues to understanding irp94 molecular features associated with extracellular stressors, including ER stress and radiation.

## 2. Results and Discussion

In preliminary experiments, the effect of various doses of radiation on BiP expression was assessed in PC12 cells by RT-PCR, and no evidence noting remarkable changes of ER chaperone BiP by *B. mori* hemolymph was found, apart from in the cell culture condition (5%) (data not shown here). Increased gene expression of BiP was observed at tested doses (5, 10, 15 Gy) and maximal induction was observed at 10 Gy of irradiation (~2–fold more than controls). Time-course studies indicated that expression of BiP showed an increase at 6 h after radiation at a dose of 10 Gy, with a peak at 24 h, and remained elevated even at 48 h post-irradiation. We also tested whether *B. mori* hemolymph directly regulated the expression of UPR molecules, and the dose-dependent effect on gene expression and induction of apoptosis according to previous data [22,23]. We also tested cell viability of *B. mori* hemolymph without radiation condition by MTT assay that no obvious changes were detected (data not shown), and we then treated healthy cells, which showed no apoptosis over the previous 7 days, with 5% hemolymph.

**Figure 1:**
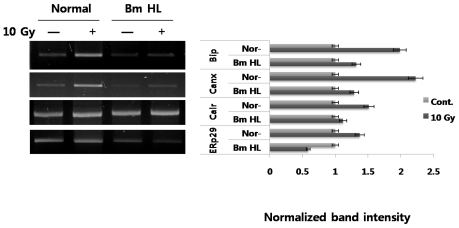
Expression of ER chaperones induced by radiation with or without the addition of silkworm hemolymph.

First, we evaluated the expression of ER chaperones induced by radiation in PC12 cells. As shown in Figure 1, radiation induced up-regulation in all tested ER chaperones (BiP and calnexin (~2-fold), calreticulin and ERp29 (~1.5-fold)) at the mRNA level. Next, we tested the additional effect of silkworm hemolymph on ER chaperone expression under radiation-irradiation conditions. Interestingly, addition of silkworm hemolymph to irradiated cells resulted in an obvious decrease in ER chaperone expression, compared with radiation alone; in the extreme case of ERp29, expression was down-regulated, compared with the control (indicated by BmHL). Need more experiments to understand that pretreatment or posttreatment of silkworm hemolymph which directly changes the basal level of UPR state.

**Figure 2:**
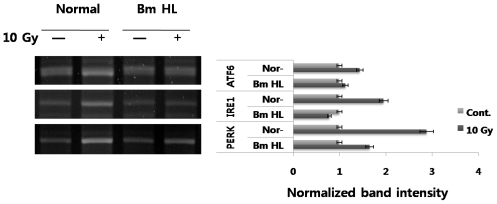
Expression of ER stress sensors induced by radiation with or without the addition of silkworm hemolymph.

UPR in mammalian cells involves three distinct ER stress sensors (IRE1, PERK, ATF6) that are downstream components of ER chaperones, and which transmit stress signals from the ER to the nucleus in response to perturbations in protein folding in the ER. While activation (autophosphorylation and dimerization) of IRE1 activates the endonuclease domains, which cleave X-box DNA-binding protein (XBP) mRNA, generating an activated form of XBP1, activation of PERK results in phosphorylation of the α subunit of eukaryotic translation initiation factor 2 (eIF2α) and inhibits translation initiation. ATF6 is cleaved at the cytosolic face of the membrane in response to ER stress, causing nuclear translocation of the N-terminal cytoplasmic domain, which contains the DNA-binding, dimerization, and transactivation domains, and subsequent binding to both ER stress-response element (ERSE) and ATF6 sites to enhance expression of ER molecular chaperone genes. We also evaluated the expression of the three ER membrane kinases in response to radiation-irradiation and the addition of silkworm hemolymph (Figure 2). As shown in Figure 1, radiation induced up-regulation in the expression of IRE1 (~2-fold), PERK (~3-fold), and ATF6 (~1.5-fold), and with the addition of silkworm hemolymph, expression of these genes showed marked decrease. *B. mori* hemolymph, specifically induced the ER stress sensors in the radiation condition as seen by transcriptional levels. However, its mechanism of action is not fully understood and needs more experiments for the XBP1 mRNA splicing, eIF2 alpha expression and ARF6 fragmentation.

Our results (Figures 1 and 2) indicated that radiation-irradiation induces up-regulation of ER chaperones, including BiP, calnexin, calreticulin, and ERp29, and ER stress sensors, including IRE1, PERK, and ATF6 at the mRNA level in PC12 cells. In particular, increased expression of ER membrane kinases suggested a strong association of stimulation by radiation with expression of ER chaperone genes and the direct ER signal transduction pathway. However, the detailed molecular mechanism underlying UPR induced by radiation will require significant study. Here, suppression of the expression of both ER chaperones and ER stress sensors by the addition of silkworm hemolymph under radiation-irradiation conditions is an interesting finding. Factors in silkworm hemolymph point to its potential for protection against extracellular-disturbance induced by radiation through down-regulation of ER stress associated proteins.

**Figure 3:**
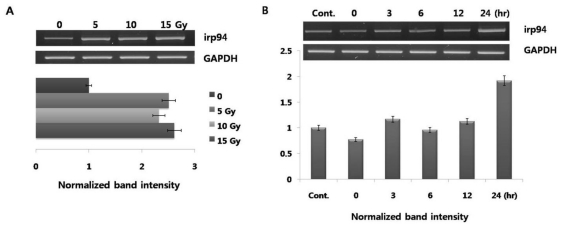
Radiation influences irp94 mRNA expression: dose- and time-dependent induction. (**A**) PC12 cells were irradiated at doses of 0, 5, 10, and 15 Gy, and total RNA was harvested; (**B**) PC12 cells were irradiated at a dose of 10 Gy and total RNA was harvested at the indicated post-irradiation times. All mRNA levels of ischemia-responsive protein 94 (irp94) were measured by semiquantitative RT-PCR. All experimental conditions were the same as those in Figure 1.

A gene encoding irp94 was first identified following characterization of a novel protein of the HSP110 family that may play an important role against ischemia. We previously demonstrated that expression of irp94 is regulated by ER stressors in neurons and thyrocytes. Although increased expression of ER chaperones (BiP, calnexin, calreticulin, ERp29) by radiation-irradiation was demonstrated in this study (Figure 1), expression of irp94 is unclear. We then performed testing to determine whether expression of irp94 was activated in PC12 cells stimulated by radiation-irradiation. Its expression showed a rapid increase in response to a radiation dose of 5 Gy (~2.5-fold; Figure 3A), and its expression was almost constant at 15 Gy. The level of increased irp94 expression by radiation is similar to those of ER chaperones (BiP and calnexin in Figure 1). When cells were irradiated with 10 Gy, the highest expression was detected after a 24 h chase (Figure 3B). This study is the first to show that radiation induced an increase in the mRNA level of irp94 in PC12 cells.

**Figure 4:**
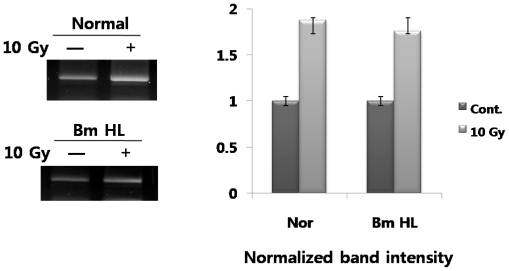
Expression of irp94 induced by radiation with or without the addition of silkworm hemolymph.

From the data above (Figure 1), while ER chaperones were up-regulated by radiation-irradiation, expression of these genes was down-regulated by the addition of silkworm hemolymph. We also performed testing to determine whether irp94 expression was induced by the addition of silkworm hemolymph after irradiation. Under these expression conditions, when cells are exposed to silkworm hemolymph, expression of ER chaperones was significantly down-regulated, as demonstrated by the results above. However, as shown in Figure 4, in contrast to expectations, irp94 expression was not obviously changed, and was not down-regulated by the addition of silkworm hemolymph after irradiation, unlike the other ER chaperones.

PC12 cells were irradiated at a dose of 10 Gy and treated with or without 5% silkworm hemolymph. Total RNA was harvested at 24 h post irradiation. All mRNA levels of immunoglobulin heavy chain binding protein (BiP), calnexin (Canx), calreticulin (Calr), and endoplasmic reticulum protein 29 (ERp29) were measured by semiquantitative RT-PCR. All experiments were performed at least three times and results represent the average. The uptake ratio (fold) is shown relative to control (1-fold). RT-PCR primers used are shown in the *Experimental Section*.

All mRNA levels of activating transcription factor 6 (ATF6), inositol-requiring enzyme 1 (IRE1), and protein kinase-like ER kinase (PERK) were measured by semiquantitative RT-PCR. All experimental conditions were the same as those in Figure 1.

PC12 cells were irradiated at a dose of 10 Gy and total RNA was harvested. All mRNA levels of irp94 were measured by semiquantitative RT-PCR. All experimental conditions were the same as those in Figure 1.

## 3. Experimental Section

### 3.1. Cell Culture and Radiation Exposure

PC12 is a cell line derived from a pheochromocytoma of the rat adrenal medulla, which is useful as a model system for neuronal experiments. PC12 cells were cultured on collagen coated flasks in 85% RPMI 1640 supplemented with 25 mM Hepes buffer, 10% heat-inactivated horse serum, and 5% heat-inactivated fetal bovine serum, 2 mM L-glutamine, 1 mM sodium pyruvate, 1 g/L d-(+)-glucose, and antibiotics: 25 μg/mL streptomycin and 25 U/mL penicillin. Cells were maintained in a humidified incubator at 37 °C in a 5% CO^2^ atmosphere. The medium was exchanged every 48 h. Cells were rinsed with 1 × PBS, pH 7.0, and detached with 0.25% trypsin/EDTA. Following centrifugation (1000 × g, 5 min), cells were sub-cultured in 25-cm^2^ flasks using a sub-cultivation ratio of 1:2 to 1:4 and were photographed every 24 h with an inverted microscope. Cells were passaged twice per week. The 80% confluent monolayer of PC12 cells was pretreated with or without 5% silkworm hemolymph for 24 h. The resulting cells were irradiated (X-ray) with a linear accelerator (Clinac 2100 C, Varian, USA) at indicated doses and times. After irradiation, the medium was immediately replaced with fresh PBS. The dishes were then returned to the incubator for another 24 h culture. Total RNA from cultured cells was extracted using an RNA isolation reagent (TRI-Reagent Ambion, Austin, USA) and used for the following RT-PCR experiments.

### 3.2. Silkworm Hemolymph Collection

Silkworm hemolymph was collected from the fifth-instar larvae of *Bombyx mori* by clipping the side of an abdominal leg. On average, 0.5 mL of hemolymph was obtained per larva. Collected hemolymph was heat-treated at 60 °C for 30 min, and then chilled and centrifuged (10,000 × g, 30 min). The supernatant was filtered through a 0.2-μ membrane filter and kept at 4 °C; 5% SH was added directly to the medium.

### 3.3. Semiquantitative RT-PCR

RT-PCR was performed using the forward primer (F) (5′–ACCACCAGTCCATCGCCATT-3′) and reverse primer (R) (5′–CCACCCTGGACGGAAGTTTG-3′) for IRE1, F (5′–AGTGGTGGCCAC TAATGGAG-3′) and R (5′–TCTTTTGTCAGGGGTCGTTC-3′) for BiP, F (5′–CTAGGCCTGGAGG CCAGGTT-3′) and R (5′–ACCCTGGAGTATGCGGGTTT-3′) for ATF6, F (5′–GGTCTGGTTCCT TGGTTTCA-3′) and R (5′–TTCGCTGGCTGTGTAACTTG-3′) for PERK, F (5′–GGGAGTCTTG TCGTGGAATTG-3′) and R (5′–TGCTTTCCAAGACGGCAGA-3′) for calnexin, F (5′–ACATCAG GAGCTAAAAGCAGCC-3′) and R (5′–TGAAACATACGTCACCCGCA-3′) for calreticulin, F (5′–CAGGATTTGCCCTATCCAGA-3′) and R (5′–GTCATTCCGTTCCTTCTCCA-3′) for irp94, F (5′–TAC AAGGTCATTCCCAAAAGCAAGT-3′) and R (5′–CGGAAGAGGTAGAAGACTGGG TAGC-3′) for ERp29, and F (5′–ACATCAAATGGGGTGATGCT-3′) and R (5′-AGGAGACAAC CTGGT CCTCA-3′) for GAPDH. RT-PCR primers were supplied from Bioneer Co. (Taejon, Korea). Unless otherwise noted, all other chemicals were purchased from Sigma (St. Louis, USA). RT-PCR conditions were as follows: 30 cycles (94 °C for 30 s, 58 °C for 30 s, and 72 °C for 1 min (with 10 min for the final cycle)) using the above primers with *Taq* DNA polymerase.

## 4. Conclusions

In summary, based on the results above, radiation-irradiation induced expression of ER chaperones, including irp94, and addition of silkworm hemolymph down-regulated expression of ER chaperone genes in irradiated cells, except irp94. We suggest that there are unknown factors in silkworm hemolymph that prevent down-regulation of irp94 expression under radiation conditions. This different molecular character for irp94 may be associated with its unique biological functions under UPR. For future studies, we propose identification and analysis of new factors from silkworm hemolymph that facilitate irp94 expression under radiation-irradiation. This will help to decipher the biological functions of irp94.

## References

[1] Schröder M, Kaufman RJ (2005). ER stress and the unfolded protein response. Mutat. Res.

[2] Ron D, Walter P (2007). Signal integration in the endoplasmic reticulum unfolded protein response. Nat. Rev. Mol. Cell Biol.

[3] Mori K (2009). Signalling pathways in the unfolded protein response: Development from yeast to mammals. J. Biochem.

[4] Schröder M (2008). Endoplasmic reticulum stress responses. Cell Mol. Life Sci.

[5] Määttänen P, Gehring K, Bergeron JJ, Thomas DY (2010). Protein quality control in the ER: The recognition of misfolded proteins. Semin. Cell Dev. Biol.

[6] Kim PS, Arvan P (1998). Endocrinopathies in the family of endoplasmic reticulum (ER) storage diseases: Disorders of protein trafficking and the role of ER molecular chaperones. Endocr. Rev.

[7] Zhang B, Wang M, Yang Y, Wang Y, Pang X, Su Y, Wang J, Ai G, Zou Z (2008). ERp29 is a radiation-responsive gene in IEC-6 cell. J. Radiat. Res.

[8] Rasheva VI, Domingos PM (2009). Cellular responses to endoplasmic reticulum stress and apoptosis. Apoptosis.

[9] Clem RJ, Koval TM (1997). Apoptosis as a Stress Response. Stress-Inducible Processes in Higher Eukaryotic Cells.

[10] Koval TM, Koval TM (1997). Stress Resistance in Lepidopteren Insect Cells. Stress-Inducible Processes in Higher Eukaryotic Cells.

[11] Choi SS, Rhee WJ, Park TH (2002). Inhibition of human cell apoptosis by silkworm hemolymph. Biotechnol. Prog.

[12] Choi SS, Rhee WJ, Park TH (2005). Beneficial effect of silkworm hemolymph on CHO cell system: Inhibition of apoptosis and increase of EPO production. Biotechnol. Bioeng.

[13] Choi SS, Rhee WJ, Kim EJ, Park TH (2006). Enhancement of recombinant protein production in Chinese hamster ovary cells through anti-apoptosis engineering using 30Kc6 gene. Biotechnol. Bioeng.

[14] Rhee WJ, Lee EH, Park JH, Lee JE, Park TH (2007). Inhibition of HeLa Cell apoptosis by storage-protein 2. Biotechnol. Prog.

[15] Tischler AS, Greene LA, Kwan PW, Slayton VW (1983). Ultrastructural effects of nerve growth factor on PC 12 pheochromocytoma cells in spinner culture. Cell Tissue Res.

[16] Hallahan DE, Spriggs DR, Beckett MA, Kufe DW, Weichselbaum RR (1989). Increased tumor necrosis factor alpha mRNA after cellular exposure to ionizing radiation. Proc. Natl. Acad. Sci. USA.

[17] Brach MA, Grub HJ, Kaisho T, Asano Y, Hirano T, Herrmann F (1993). Ionizing radiation induces expression of interleukin 6 by human fibroblasts involving activation of nuclear factor-kappa B. J. Biol. Chem.

[18] Brach MA, Hass R, Sherman ML, Gunji H, Weichselbaum R, Kufe D (1991). Ionizing radiation induces expression and binding activity of the nuclear factor kappa B. J. Clin. Invest.

[19] Yagita Y, Kitagawa K, Taguchi A, Ohtsuki T, Kuwabara K, Mabuchi T, Matsumoto M, Yanagihara T, Hori M (1999). Molecular cloning of a novel member of the HSP110 family of genes, ischemia-responsive protein 94 kDa (irp94), expressed in rat brain after transient forebrain ischemia. J. Neurochem.

[20] Kim SW, Yoo IS, Koh HS, Kwon OY (2001). Ischemia-responsive protein (irp94) is up-regulated by endoplasmic reticulum stress. Z. Naturforsch C.

[21] Kim SW, Chung SP, Kim SH, Choi JS, Kwon K, Kwon OY (2007). Ischemia-responsive protein (irp94) gene expression in neurons. Z. Naturforsch C.

[22] Rhee WJ, Kim EJ, Park TH (1999). Kinetic effect of silkworm hemolymph on the delayed host cell death in an insect cell-baculovirus system. Biotechnol. Prog.

[23] Rhee WJ, Kim EJ, Park TH (2002). Silkworm hemolymph as a potent inhibitor of apoptosis in Sf9 cells. Biochem. Biophys. Res. Commun.

